# EEG-Based Characterization of Learning Potential-Related Neural Representations via Dynamic Assessment Tasks

**DOI:** 10.3390/jintelligence14090194

**Published:** 2026-09-01

**Authors:** Jingying Chen, Tengfei Gao, Rui Li, Zhiyi Yang, Yuhan Li

**Affiliations:** 1 College of Educational Science, Kashi University, Kashi 844000, China; 2 National Engineering Research Center of Educational Big Data, Central China Normal University, Wuhan 430079, China; 3 School of Foreign Languages, Central China Normal University, Wuhan 430079, China

**Keywords:** learning potential, EEG, dynamic learning paradigm, assessment methods, SHapley additive explanations

## Abstract

Learning potential assessment is important for understanding individual differences in learning processes and cognitive development. Traditional assessments mainly rely on questionnaires or behavioral data collected during tasks, emphasizing external performance while lacking sensitivity to learners’ internal cognitive activities. Therefore, they provide limited evidence about the neurocognitive mechanisms underlying individual learning potential. This study investigated whether task-evoked EEG responses acquired during a dynamic learning paradigm could characterize individual differences in task-specific learning performance under graduated instructional support. Participants were 50 undergraduate students who completed a dynamic cognitive task designed to evaluate learning performance under graduated instructional support. An electroencephalogram (EEG)-based Multi-scale Learning Potential Assessment (MS-LPA) framework was developed, including a Dynamic Learning Paradigm with progressively complex cognitive tasks and responsive cueing; multiscale Neural Complexity Representations quantifying signal complexity and inter-regional synchronization; and a ResNet-18 model with SHAP-based interpretability for classification and biomarker identification. Experiments showed that MS-LPA captured task-evoked neural responses and outperformed traditional outcome-driven evaluation schemes. Entropy-based features achieved an accuracy of 0.77, and functional connectivity matrices reached 0.83. These findings demonstrate the feasibility of using EEG-derived neural representations to differentiate individual differences in task-specific dynamic learning performance and provide insights into its underlying neural mechanisms.

## 1. Introduction

The identification and assessment of learning potential have become important research topics at the intersection of psychology, education, and neuroscience. According to Vygotsky’s theory of the Zone of Proximal Development, potential refers to the maximum level of development that an individual can achieve with appropriate support (Vygotsky, 1978). Building on this view, learning potential can be understood as an individual’s latent capacity to acquire new knowledge, skills, or cognitive strategies under proper guidance or scaffolding. Unlike static achievement indicators, learning potential emphasizes the dynamic and future-oriented aspects of cognitive development (Lauchlan & Elliott, 2001). Learning potential is reflected not only by whether a learner eventually reaches the correct solution but also by the degree to which performance can be modified through graduated instructional mediation. From this perspective, the amount of support required to achieve successful task completion provides an indirect indicator of cognitive modifiability, which constitutes a central component of learning potential in dynamic assessment theory (Lauchlan & Elliott, 2001; Sternberg & Grigorenko, 2002).

Despite the effectiveness of current learning potential assessments in characterizing individual developmental levels, they fundamentally rely on standardized static tests, such as questionnaires or performance-based behavioral evaluations, which emphasize an individual’s ability at a fixed outcome point (Jensen, 1998; McGrew, 2009). While these approaches are useful in certain assessment or screening contexts, they predominantly reflect “transient snapshots” of cognitive functioning rather than the underlying mechanisms or adaptability of the learner. That is, such static paradigms inherently neglect the dynamic, context-sensitive nature of learning (Zatorre et al., 2012), and more critically, fail to capture neural and cognitive responses elicited during dynamic learning tasks (Blume et al., 2019). This perspective has prompted a growing paradigm shift from outcome-based ability measurement toward process-oriented potential exploration (Sun et al., 2024). In contrast, neuroscience-driven methods, such as electroencephalography (EEG), offer a powerful avenue for characterizing the neural correlates of task-specific learning performance during dynamic assessment (Beauchemin et al., 2024). Recent studies have unveiled EEG-based neural correlates of learning capacity and cognitive flexibility, suggesting that learning-related neural responses vary according to task demands and instructional support during dynamic assessment (Dimitriadis et al., 2015).

Developing EEG-based methods to characterize interpretable neural patterns during dynamic assessment is therefore crucial for precise and individualized assessment of learning potential. Compared with traditional evaluations, EEG-based approaches can characterize neural responses elicited during cognitively demanding learning tasks. With its high temporal resolution, EEG provides a unique window for capturing temporally localized neural responses and characterizing learning-related changes in functional connectivity and neural complexity (Gkintoni et al., 2025). However, existing research has not fully realized the potential of EEG for dynamic learning potential assessment. Several challenges remain: (1) designing valid task paradigms that reliably elicit learning-related neural states and individual differences; (2) extracting quantifiable neural features associated with learning-related cognitive processes and cognitive processes; and (3) improving the interpretability of the relationship between dynamic brain activity and learning potential.

To address these challenges, this study proposes an EEG-based Multi-scale Learning Potential Assessment (MS-LPA) framework for interpretable assessment of learning potential. Grounded in high-temporal-resolution EEG analysis, MS-LPA is designed to characterize task-evoked neural responses during dynamic assessment. It should be noted that the objective of the present study is to characterize participant-level neural representations associated with dynamic assessment, rather than to explicitly model the continuous temporal evolution of learning trajectories.The framework consists of three core modules:

• Dynamic Learning Paradigm: A continuous task structure involving reading comprehension, responsive cueing, and error-based revision is employed to elicit task-related neurocognitive responses and obtain learning potential scores during active learning (Section 4.1).
• Multiscale Neural Complexity Representations: Entropy-based and functional connectivity metrics are extracted to characterize signal complexity and inter-regional neural coordination during learning, providing a multiscale characterization of task-evoked brain activity (Section 4.2).
• Interpretable Deep Learning Model: A ResNet-18 model with SHAP-based interpretability is used to map EEG features to learning potential classifications and identify critical neural biomarkers (Section 4.3).

Experiments were conducted on an EEG dataset collected from 50 participants during dynamic English reading comprehension tasks. The proposed MS-LPA was evaluated in terms of: (1) the effectiveness of the dynamic learning paradigm in eliciting representative neurocognitive responses; (2) the capability of multiscale neural complexity representations to characterize task-specific learning performance across frequency bands; and (3) the ability of interpretable deep learning to identify frequency- and region-specific neural biomarkers.

The main contributions of this study are summarized as follows:

- Design an EEG-based dynamic task paradigm that captures task-evoked neural responses during dynamic assessment and overcomes the limitations of external behavioral outcome evaluations.
- Propose a unified neural representation integrating complexity and connectivity features, providing a multiscale neural representation associated with task-specific learning performance from both local and large-scale brain dynamics.
- Achieve accurate and interpretable learning potential assessment, addressing the transparency limitations of existing black-box models.

## 2. Related Work

This section reviews key developments in EEG-based learning potential assessment, focusing on two primary methodological approaches: (1) emerging assessment strategies that shift from static, outcome-based assessments to dynamic, process-oriented frameworks; (2) EEG-based modeling of cognitive processes using neurophysiological indicators. These paper streams collectively underpin the motivation for our proposed MS-LPA framework, which integrates dynamic task EEG analysis with interpretable modeling.

### 2.1. From Outcome-Based Testing to Process-Oriented Potential Assessment

Traditionally, learning potential has been inferred from standardized, fixed time point tests such as questionnaires or performance-based tasks administered at discrete time points (Jensen, 1998). However, these snapshot-like assessments tend to reflect one-time performance rather than continuous cognitive evolution, and fail to account for how cognitive strategies adapt and evolve during the learning process (Blume et al., 2019). This methodological constraint has motivated a shift toward process-oriented models that treat potential as a dynamic capacity rather than a fixed trait (Sun et al., 2024).

Emerging work in educational neuroscience and process-based assessment theory highlights the need to shift the evaluation lens from external behavioral performance to dynamic neural processes. This includes measuring how individuals adjust their strategies, manage cognitive load, and coordinate brain resources throughout learning tasks. For example, Vygotsky’s “Zone of Proximal Development” emphasizes that potential is best measured through performance under assistance, rather than isolated task scores.

Meanwhile, recent research emphasizes the need for continuous task paradigms that elicit cognitively meaningful transitions in brain activity. For instance, studies have employed reading comprehension with dynamic cueing or error-based revision to induce variations in attentional load and memory retrieval demands, thereby enabling the observation of learning-related EEG changes across time (Yang & Hu, 2024).

### 2.2. EEG-Based Modeling of Learning Processes and Learning Potential

EEG provides a non-invasive method with high temporal resolution for investigating dynamic neural processes underlying human learning. Its sensitivity to rapid brain activity fluctuations makes it suitable for assessing learning-related cognitive operations, such as attention, working memory, and problem solving (Jawed et al., 2024). Traditional EEG analyses have mainly focused on static indicators, including event-related potentials and spectral power, which provide limited insight into evolving neural responses to cognitive demands.

Recent studies have shifted toward modeling more complex and temporally dynamic EEG features. Entropy-based metrics, such as sample entropy and permutation entropy, have been used to quantify neural signal complexity and reflect the adaptability and efficiency of neural systems during learning (Nayani et al., 2025). These features indicate how flexibly the brain responds to novel or increasing task demands, which is closely related to learning potential (Naeini & Duvall, 2012).

Functional connectivity analyses further capture coordination across distributed brain regions. Metrics such as coherence, phase synchronization, and phase-locking value are commonly used to characterize neural reorganization during learning (Liu et al., 2025). Compared with localized EEG measures, these network-level properties provide richer information about individual cognitive capacity.

Multi-band EEG studies have also shown that different oscillatory activities support distinct cognitive functions. For example, theta-gamma coupling and prefrontal synchrony have been associated with memory encoding and cognitive control (Scharinger et al., 2023), while alpha-band suppression has been linked to attentional efficiency during high-load tasks (Klimesch, 2012). Variability in theta and gamma phase locking may also reflect adaptive reallocation of cognitive resources (Papaioannou et al., 2022).

Despite these advances, few studies have operationalized process-based learning potential assessment using neurophysiologically grounded and interpretable methods. Existing models often rely on black-box algorithms with limited transparency, reducing their educational utility. This study addresses this gap by introducing a dynamic task EEG-based framework to model evolving neurocognitive states during learning and link neural features with learning potential classifications.

## 3. Experiments

**Figure 1:**
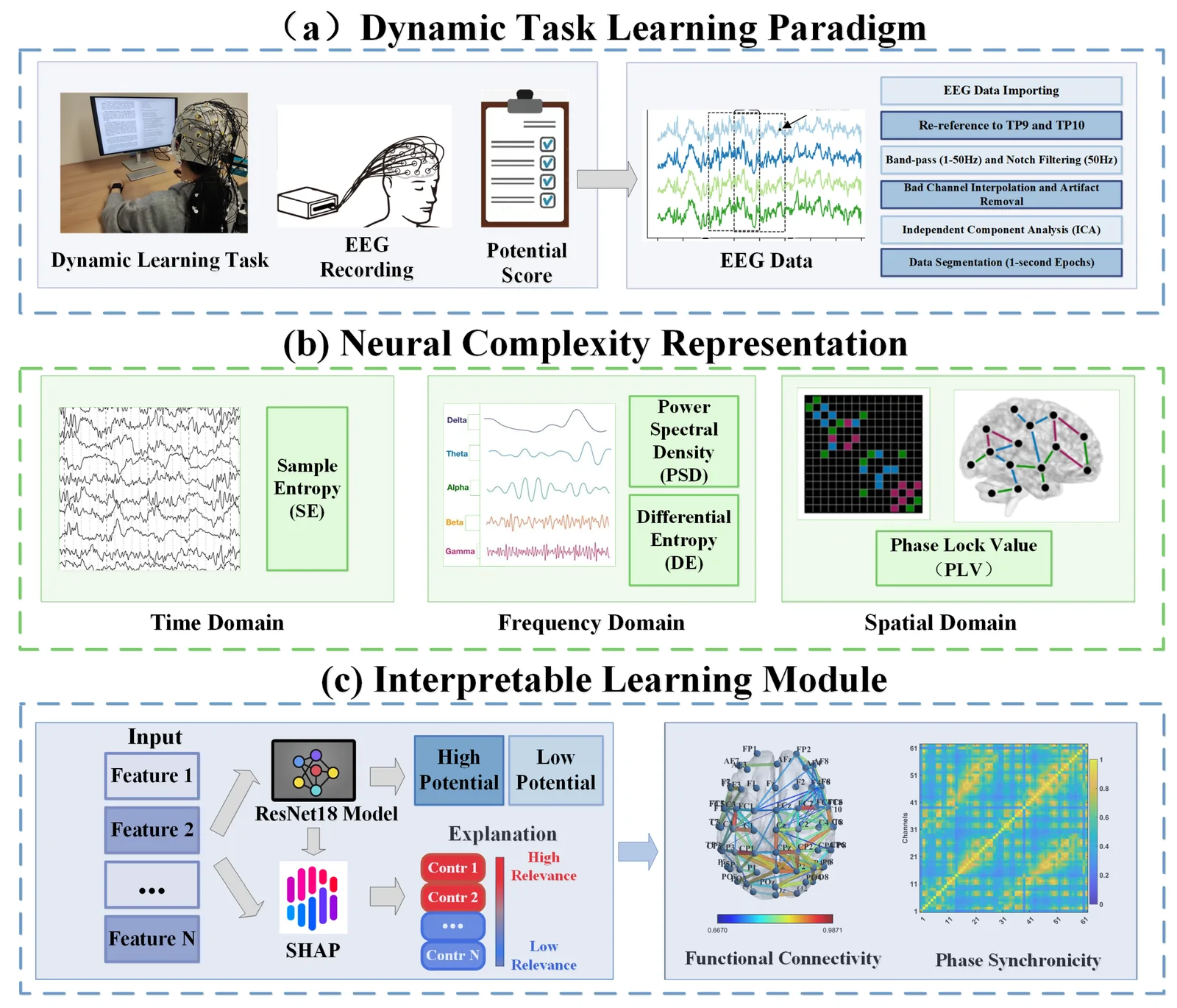
Overview of the proposed EEG-based Multi-scale Learning Potential Assessment (MS-LPA) framework. The pipeline consists of three stages: (**a**) Dynamic Task Learning Paradigm, in which EEG data and learning-potential scores are collected during dynamic tasks; (**b**) Neural Complexity Representation, in which EEG signals are transformed into entropy-based features and functional connectivity metrics to characterize neurocognitive states; and (**c**) Interpretable Learning Module, in which a ResNet-18 model with SHAP-based interpretability classifies learning potential and identifies reliable neurobiomarkers.

This study is carefully designed to assess participants’ learning potential and neurocognitive states through the EEG-based Multiscale Learning Potential Assessment (MS-LPA) Framework. As illustrated in Figure 1, the complete evaluation pipeline consists of five sequential steps and addresses three fundamental objectives: **designing** a dynamic task learning paradigm, **capturing** neurocognitive features of learning potential, and **leveraging** these features as interpretable neurobiomarkers. Specifically:

1. EEG acquisition during the dynamic task.
2. Preprocessing of EEG signals.
3. Extraction of multiple EEG features, including Spectral Entropy (SE), Power Spectral Density (PSD), Differential Entropy (DE), and Phase-Locking Value (PLV).
4. Classification of learning potential levels using a ResNet-18 deep learning model with SHAP for interpretability.
5. Identification of EEG-based biomarkers.

### 3.1. Experimental Paradigm

This study was designed to evaluate participants’ neurocognitive states under different levels of learning potential. Grounded in Vygotsky’s Zone of Proximal Development (ZPD), the paradigm adopted a dynamic English reading comprehension task as the core assessment approach. Materials were selected from past CET-4 and CET-6 papers in China, which involve lexical access, syntactic parsing, and discourse-level inference, thereby providing a cognitively demanding context for evaluating learning potential (Jiang, 2020).

The dynamic assessment paradigm was designed to evaluate learning potential by capturing real-time neural responses and behavioral adaptations during task engagement. It included three stages:

1. Initial Assessment: the first attempt at answering reading comprehension questions.
2. Dynamic Prompt Selection: prompt selection following incorrect answers.
3. Reassessment: the second attempt after receiving the selected prompt.

If a participant answered incorrectly during the initial attempt, the system provided prompts at different difficulty levels. Participants selected a prompt level before reattempting the question, providing an implicit measure of learning potential through their feedback use and strategic control.

Prompt types were defined as follows:

1. Low-level Prompt (n=1): a minimal cue, such as locating relevant text or suggesting synonym replacement.
2. Medium-level Prompt (n=2): a moderate cue, such as pointing to a specific paragraph or sentence with a brief explanation.
3. High-level Prompt (n=3): a detailed cue, such as indicating the exact sentence or providing an explicit explanation.

Each prompt level was associated with a different scoring rule, encouraging strategic decisions based on task performance. This adaptive process simulates real-world learning and enables objective profiling of learning potential by observing how individuals respond to feedback and support under cognitive demand.

### 3.2. Experimental Procedure

**Figure 2:**
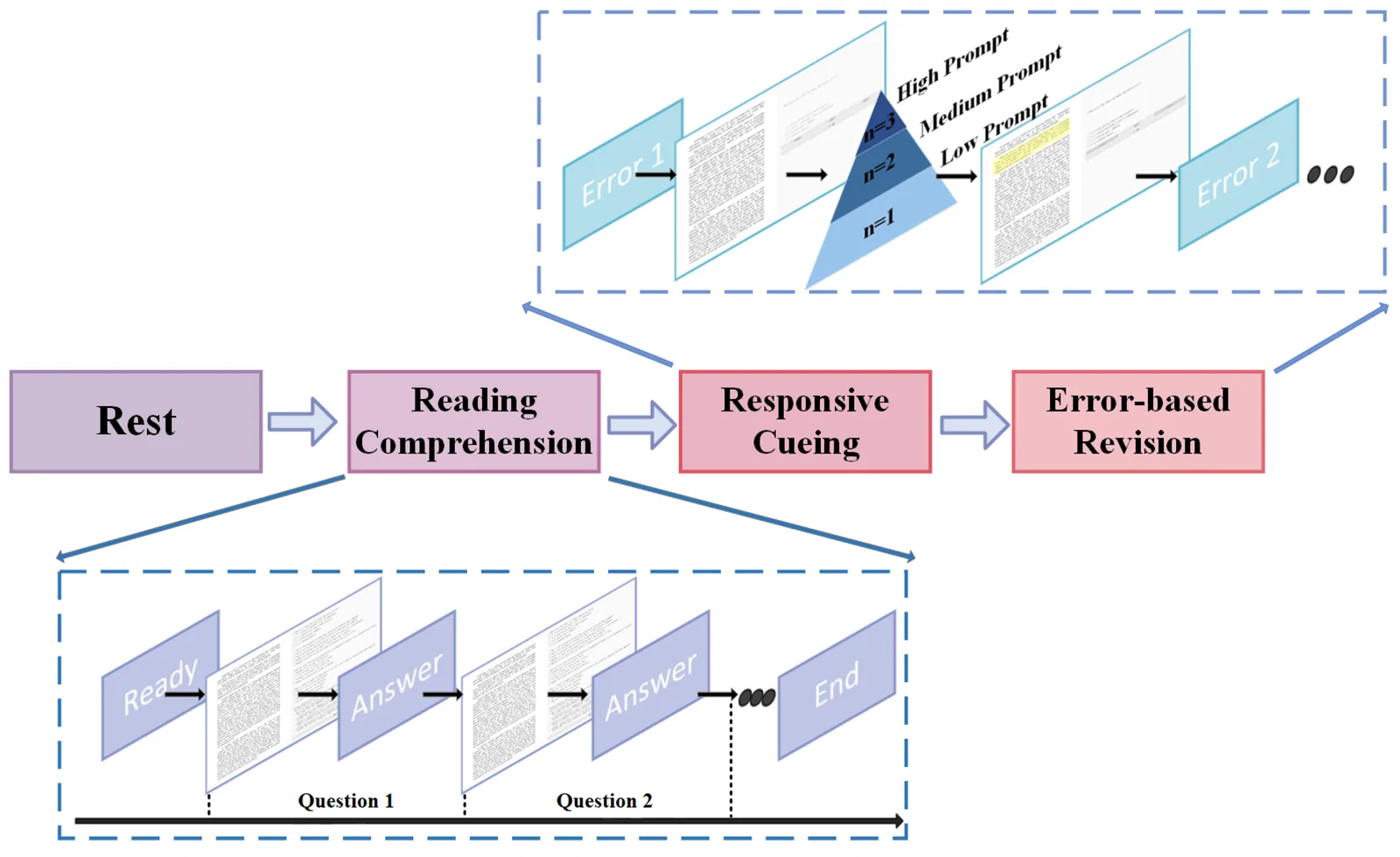
Detailed trial procedure illustrated for an example participant. During the assessment, each reading passage was accompanied by multiple questions that were answered sequentially. When an incorrect response occurred, a responsive cueing procedure was initiated, providing graduated instructional prompts (low-, medium-, and high-level prompts) according to the participant’s performance. Participants then revised their answers based on the provided cues. The cueing-revision process was repeated until the correct response was obtained or all prompt levels had been exhausted.

Participants followed a structured sequence during the assessment as shown in Figure 2. Before the formal task, a 2-min resting-state EEG recording was conducted to establish a baseline for subsequent neural signal analysis. Upon completion of the resting period, an auditory cue was automatically delivered by the assessment system to indicate the onset of the experimental session. Participants were required to complete the entire dynamic assessment procedure, which included initial testing, error-triggered hint selection, and repeated response phases. EEG recording was terminated upon task completion. The system then automatically computed task performance metrics, including reading score, hint selection behavior, and error rates. Based on the concept of mediational scoring, a learning potential score was subsequently calculated, reflecting the participant’s dynamic learning capacity under cognitive load.

### 3.3. Learning Potential Score

To characterize individual differences in performance under graduated instructional support, we introduced a Learning Potential Score (LPS). Rather than representing a pure measure of learning capacity independent of prior knowledge, the LPS was designed as a task-specific dynamic learning index that jointly reflects participants’ initial task performance and the amount of assistance required to obtain a correct response.

Each question had a maximum mediated score of 5. A correct response on the first attempt was awarded 5 points, indicating successful task completion without external assistance. If the initial response was incorrect, subsequent attempts received progressively lower scores according to the level of support required: 4 points for a correct response on the subsequent attempt without an additional prompt, 3 points following a low-level prompt, 2 points following a medium-level prompt, 1 point following a high-level prompt, and 0 points if the response remained incorrect after all prompts. Accordingly, higher scores indicated better independent performance and/or successful task completion with less instructional assistance.

The LPS was calculated as follows:

(1) $$\mathrm{LPS}=\frac{2\;\mathrm{MediatedScore}-\mathrm{ActualScore}}{\mathrm{MaxScore}},$$

where MediatedScore denotes the sum of the scores obtained across the initial and mediated response stages, ActualScore denotes the raw score obtained before instructional support, and MaxScore denotes the maximum attainable score. Because the scoring procedure rewards both correct initial performance and successful responses obtained with limited assistance, the LPS should be interpreted as an index of task-specific dynamic learning performance rather than as a measure of learning potential that is completely independent of baseline English proficiency, reading comprehension, or test-taking ability.

To examine the relationship between EEG dynamics and task-related learning performance, participants were divided into high- and low-potential groups using the median LPS value of 0.75. Participants with LPS values greater than or equal to the median were assigned to the high-potential group, whereas those with values below the median were assigned to the low-potential group. This procedure resulted in a relatively balanced distribution, with 24 participants in the high-potential group and 26 participants in the low-potential group. The median split was used solely to construct binary labels for classification and does not imply that dynamic learning performance is inherently categorical. EEG features were subsequently used as input variables to train a classification model for distinguishing the two LPS groups. The resulting classification performance was therefore interpreted as reflecting neural differences associated with task-specific dynamic learning performance under mediated English reading conditions, rather than as direct evidence of a domain-general or ability-independent measure of learning potential.

### 3.4. Participants

Fifty undergraduate students and graduate students (20 men and 30 women) participated in this potential assessment experiment. All of them are native Chinese speakers, all right-handed, and have no mental disease or color vision disorders. Participants were between the ages of 20 and 25 (Mean = 22, STD = 1.92). Before the start of the potential assessment experiment, participants are required to understand the experimental process, and sign an informed consent document. The study protocol is approved by the Institutional Review Board (CCNU-IRB-202312043b).

## 4. Methodology

### 4.1. Dynamic Task Learning Paradigm

EEG data were collected in an electromagnetically shielded chamber at Central China Normal University. A 64-channel EEG system based on the international 10–20 layout (Brain Products platform) was used, with a sampling rate of 1000 Hz. Electrode impedance was maintained below 5kΩ throughout the recording.

EEG preprocessing was conducted using MATLAB R2020a and EEGLAB (Delorme & Makeig, 2004). The raw signals were re-referenced to the average of TP9 and TP10 electrodes. A 1–50 Hz band-pass filter and a 50 Hz notch filter were applied to remove low-frequency drift, high-frequency noise, and power-line interference. Bad channels were interpolated, and independent component analysis (ICA) was used to remove ocular and muscular artifacts. The preprocessed EEG data were segmented into 1-s sliding windows and decomposed into five canonical frequency bands: delta (δ: 1–4 Hz), theta (θ: 4–8 Hz), alpha (α: 8–13 Hz), beta (β: 13–30 Hz), and gamma (γ: 30–50 Hz).

### 4.2. Neural Complexity Representation

EEG signals are non-stationary and stochastic, making direct analysis of raw signals limited in interpretability. Therefore, features were extracted from temporal, spectral, and spatial domains to characterize neural responses under different learning potential states.

**(1) Temporal Domain.** Sample Entropy (SE) was used to quantify the temporal complexity of EEG signals by measuring the probability of new pattern emergence in a time series (X. Li et al., 2016). SE is defined as:

(2) $$\mathrm{SampEn}\left(m,r\right)=-\ln\left(\frac{B^{m+1}\left(r\right)}{B^{m}\left(r\right)}\right),$$

where $B^{m}\left(r\right)$ denotes the proportion of similar vector pairs within threshold *r* in the *m*-dimensional space.

**(2) Spectral Domain.** Power Spectral Density (PSD) characterizes the distribution of signal power across frequencies and provides information about the spectral characteristics of EEG activity (D. Li et al., 2022). To capture the time-varying spectral properties of EEG signals, the short-time Fourier transform (STFT) was first applied using a sliding Hann window to reduce spectral leakage. For the *i*-th EEG channel, the STFT was defined as:

(3) $$X_{i}\left(t,f\right)=\int_{-\infty }^{+\infty }x_{i}\left(u\right)w\left(u-t\right)e^{-j2\pi fu}\;du,$$

where $x_{i}\left(u\right)$ denotes the EEG signal of the *i*-th channel and w(u−t) denotes the time-shifted window function.

The corresponding PSD was then estimated from the normalized squared magnitude of the STFT coefficients:

(4) $$S_{i}\left(t,f\right)=\frac{|X_{i}{\left(t,f\right)|}^{2}}{f_{s}\sum_{n=0}^{L-1}w^{2}\left[n\right]},$$

where $f_{s}$ denotes the sampling frequency, w[n] is the Hann window, and *L* denotes the window length.

For each EEG channel and frequency band, the band-specific PSD feature was obtained by averaging the PSD values across time windows and frequency bins within the corresponding band:

(5) $$\mathrm{PSD}\left(i,j\right)=\frac{1}{N_{t}N_{f}^{\left(j\right)}}\sum_{t=1}^{N_{t}}\sum_{f\in B_{j}}S_{i}\left(t,f\right),$$

where *i* (i=1,2,…,64) denotes the EEG channel, *j* (j=1,2,…,5) denotes the frequency band, $B_{j}$ represents the set of frequency bins within the *j*-th band, $N_{t}$ is the number of time windows, and $N_{f}^{\left(j\right)}$ is the number of frequency bins in that band.

**(3) Spatial Domain.** To characterize inter-regional brain connectivity, Phase Locking Value (PLV) was used to quantify phase synchronization between EEG channels (Dvorak et al., 2018). PLV is defined as:

(6) $$\mathrm{PLV}=\left|\frac{1}{N}\sum_{i=0}^{N-1}e^{j\left[\varphi_{x}\left(i\Delta t\right)-\varphi_{y}\left(i\Delta t\right)\right]}\right|,$$

where Δt is the sampling period, *N* is the number of samples, and $\varphi_{x}\left(t\right)$ and $\varphi_{y}\left(t\right)$ denote the phases of signals x(t) and y(t), respectively.

### 4.3. Interpretable Learning Module

**Figure 3:**
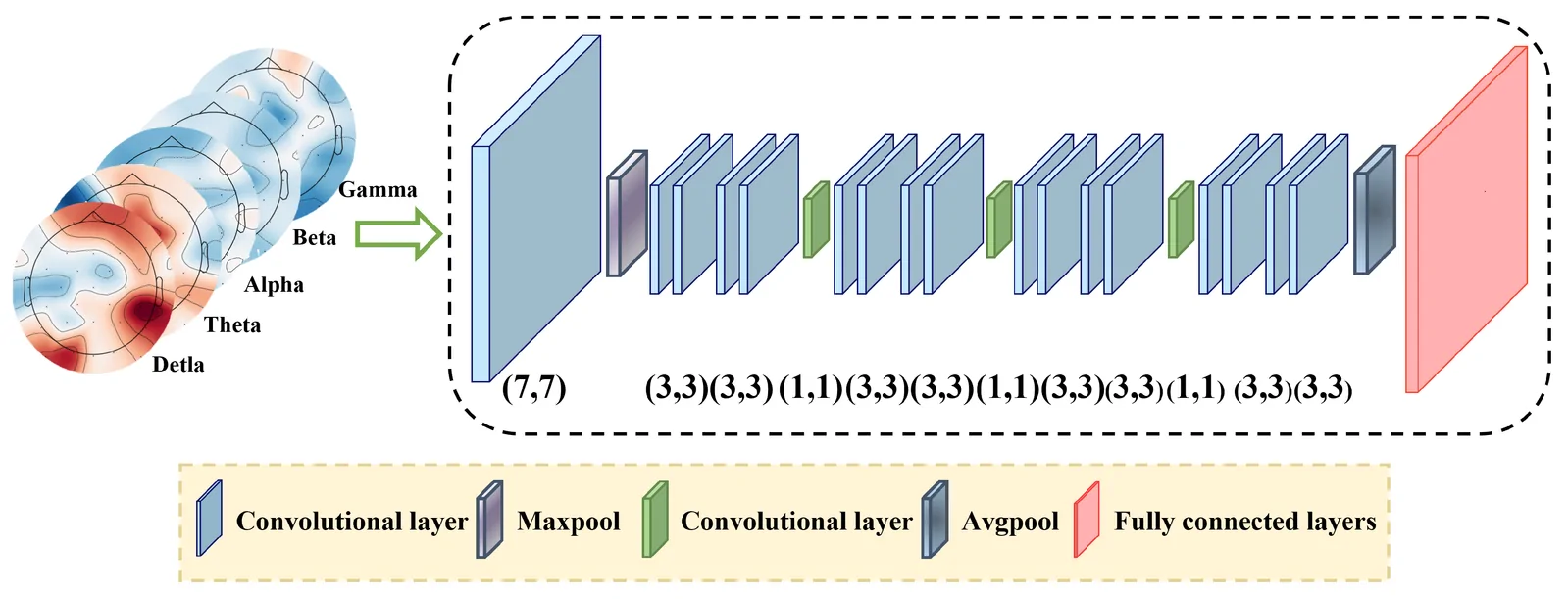
The structure of the learning potential recognition model. The input consists of EEG feature maps derived from five frequency bands (Delta: 1–4 Hz, Theta: 4–8 Hz, Alpha: 8–13 Hz, Beta: 13–30 Hz, and Gamma: 30–50 Hz), which are used independently for model training and evaluation. The network follows the standard ResNet-18 architecture, including an initial 7×7 convolution, successive residual blocks composed of 3×3 convolutions, 1×1 convolutional shortcuts for feature projection, max-pooling, global average pooling, and a fully connected layer for classification.

To distinguish individuals with high and low learning potential, we employed a ResNet-18 architecture (Du et al., 2022). ResNet-18 incorporates residual connections that alleviate the vanishing gradient problem and support the extraction of both low-level and high-level EEG representations. This structure is suitable for EEG analysis because learning-related signals often contain informative low-frequency patterns together with high-frequency noise and subtle temporal fluctuations. The residual connections help preserve low-level neural information while enabling deeper layers to learn higher-order temporal and spatial features associated with learning potential. The detailed architecture is shown in Table 1. Before classification, the extracted EEG features were transferred into feature matrices as shown in Figure 3. Specifically, the SE, PSD, and DE features were represented as channel–batch matrices (n×62), while PLV was represented as functional connectivity matrices (n×62×62). Each feature representation was independently normalized and converted into the input format required by ResNet-18. Each EEG feature type was separately used as the input to the same ResNet-18 architecture, with exactly the same parameters and configuration. In addition, to improve model interpretability, DeepSHAP was employed to quantify the contribution of each EEG feature to the classification decision. SHAP values were computed based on the trained neural network model, and the feature importance was obtained by averaging the absolute SHAP values across all test samples. The resulting SHAP values reflect the predictive importance of features within the trained model and were used to interpret which EEG representations contributed most to distinguishing the two LPS groups.

During training, the model uses the Adam optimizer with a weight decay of 0.03. The initial learning rate is set to 0.001 and is adjusted using a cosine annealing learning rate scheduler with a warm-up strategy. The batch size is set to 128, and the model is trained for 50 epochs. All executables for the experiments were implemented in a PyTorch 2.6.0 environment on a workstation running Windows, equipped with an NVIDIA GeForce RTX 4060 GPU, 32 GB RAM, and an Intel Core i7-13700KF CPU. A leave-one-subject-out cross-validation (LOSO-CV) protocol is adopted for performance evaluation. Each full round of LOSOCV contains 50 folds, with one participant held out as the test set and all remaining participants used for training in each fold.

### 4.4. Statistical Analyses

Statistical analyses were performed using SPSS 27.0 and Python 3.9. The normality of behavioral and EEG variables was assessed using the Shapiro-Wilk test (Z<1.96, p<0.05). Variables satisfying normality assumptions were compared using two-tailed independent-sample *t*-tests, statistical significance was set at p<0.05. To account for multiple comparisons across EEG features and frequency bands, the Benjamini–Hochberg false discovery rate (FDR) correction was applied. Statistical significance was determined using the FDR-corrected *p*-values (q < 0.05). Participants were divided into high- and low-learning-potential groups according to the median value of the LPS. The high-potential group included 24 participants, while the low-potential group included 26 participants. No significant differences were observed between groups in age (high-potential: 22.14 ± 1.85 years; low-potential: 21.86±2.01 years; *t* = 0.51, *p* = 0.61).

## 5. Results

The results focused on evaluating the effectiveness of the proposed MS-LPA: (1) validating the advantages of the dynamic learning paradigm in eliciting neurocognitive features of learning potential compared to traditional paradigms; (2) assessing the performance of multiscale neural complexity representations across different potential levels; and (3) evaluating the SHAP-ResNet18 model in terms of its effectiveness in capturing potential-related neural patterns while ensuring high prediction accuracy and interpretability.

### 5.1. Classification Performance of EEG Features in Learning Potential Discrimination

To evaluate the discriminative capacity of EEG-derived features, five feature types, including raw signals, DE, PSD, SE, and PLV, were tested using ResNet-18 under the leave-one-subject-out cross-validation (LOSO-CV) strategy. The classification results across all feature-band combinations are summarized in Table 2.

PLV consistently achieved the best performance across frequency bands, with accuracies ranging from 0.77 to 0.83. The highest accuracy was obtained in the Beta band (0.83 ± 0.05), indicating that inter-regional phase synchrony provides highly discriminative information for learning potential classification.

In comparison, raw EEG signals showed limited performance, especially in the Gamma band (0.49 ± 0.20). PSD features achieved relatively stable results in the Beta (0.77 ± 0.11), Gamma (0.73 ± 0.19), and Alpha (0.72 ± 0.16) bands, while DE reached its best performance in the Theta band (0.77 ± 0.11). SE produced the lowest overall performance, with a peak accuracy of 0.70 ± 0.19. Overall, these results demonstrate that functional connectivity features, particularly Beta-band PLV, are the most effective EEG predictors for distinguishing learning potential.

### 5.2. Validation of the Dynamic Learning Paradigm

This experiment evaluated whether the proposed dynamic learning paradigm could effectively capture individual differences in learning potential by examining behavioral adaptation and neural responses during the learning process. Behavioral indicators and EEG features showing significant group differences were analyzed between high- and low-potential groups.

**Figure 4:**
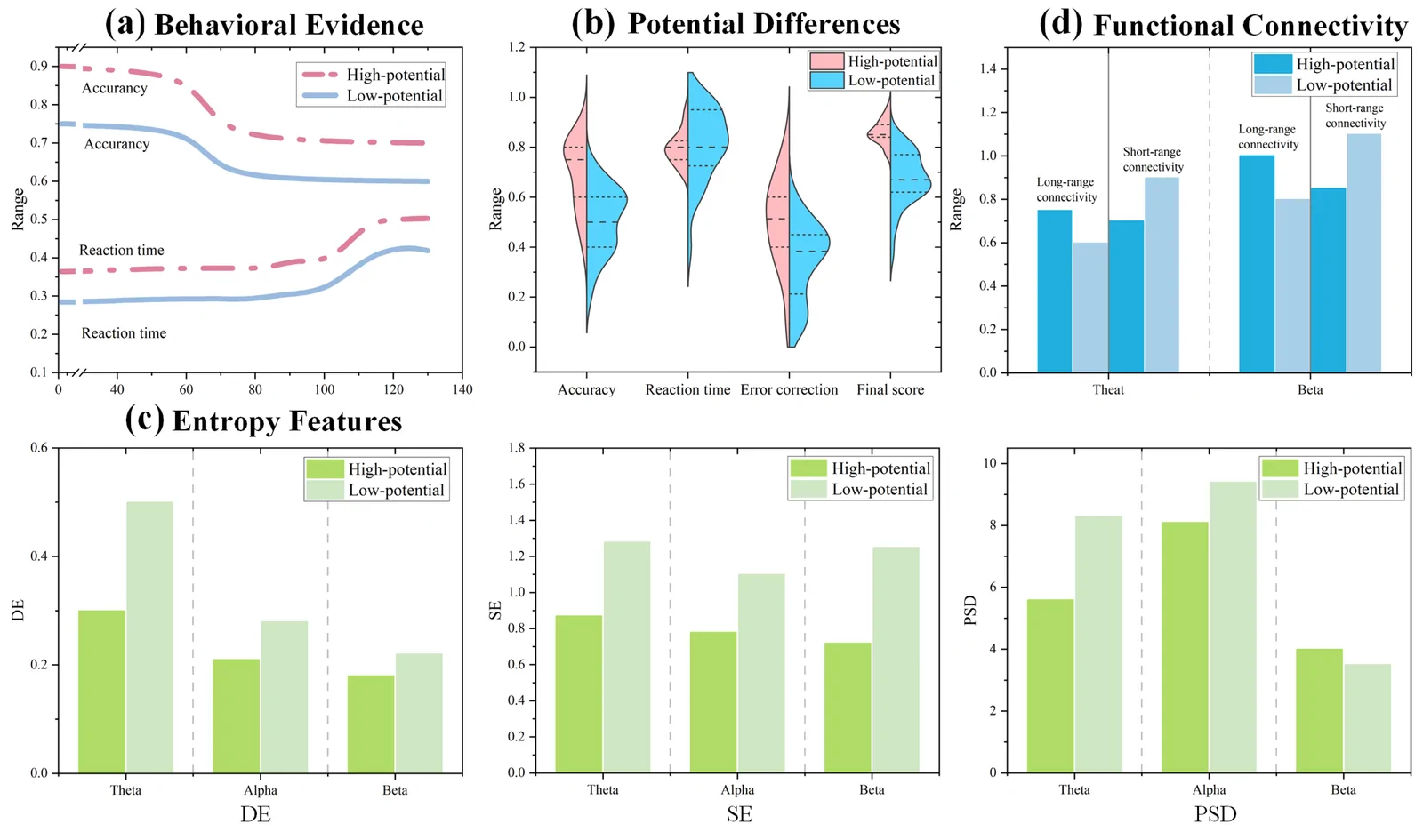
Validation of the Dynamic Learning Paradigm: (**a**) Behavioral evidence of the Dynamic Learning Paradigm; (**b**) Task performance differences across potential groups; (**c**) Patterns of entropy features and (**d**) Functional connectivity performance.

**(1) Behavioral Evidence of the Dynamic Learning Paradigm:** As shown in Figure 4a, task difficulty progressively increased during the dynamic assessment, accompanied by a gradual decrease in accuracy and changes in response time. These behavioral changes differed across the two groups. The high-potential group maintained relatively higher accuracy throughout the task, whereas the low-potential group showed a more pronounced decline as task demands increased. In contrast, the overall reaction times of the two groups were relatively comparable, although greater variability was observed in the low-potential group. The demographic characteristics and overall behavioral performance of the two groups are summarized in Table 3.

Statistical comparisons of the behavioral measures are further presented in Figure 4b. The high-potential group achieved significantly higher accuracy than the low-potential group (*t* = 3.6, $p_{\mathrm{FDR}}=0.03$, Cohen’s *d* = 0.9). The overall reaction times of the two groups were relatively comparable, although greater variability was observed in the low-potential group. (*t* = 0.36, Cohen’s *d* = 0.5). Furthermore, the high-potential group demonstrated superior error correction ability after receiving prompts (*t* = 2.9, $p_{\mathrm{FDR}}=0.05$, Cohen’s *d* = 0.72) and achieved higher final scores (*t* = 4.1, $p_{\mathrm{FDR}}=0.01$, Cohen’s *d* = 1.1). These findings indicate that the dynamic paradigm captured individual differences in task performance, feedback utilization, and adaptive learning across the assessment process.

**(2) Neural Complexity Differences Evoked by the Dynamic Paradigm:** For entropy-based features (Figure 4c), significant group differences were mainly observed in the theta band. Compared with the high-potential group, the low-potential group exhibited higher theta-band DE and SE values ($p_{\mathrm{FDR}}=0.01$, Cohen’s *d* = 0.81), indicating increased neural irregularity and greater cognitive processing demands during dynamic learning. Similar but weaker differences were observed in alpha-band entropy features ($p_{\mathrm{FDR}}=0.05$, Cohen’s *d* = 0.43). PSD analysis further revealed frequency-dependent differences between groups, with the most prominent differences occurring in the theta band. The high-potential group showed reduced theta-band power compared with the low-potential group ($p_{\mathrm{FDR}}=0.05$, Cohen’s *d* = 0.71), suggesting more efficient neural resource allocation during task engagement.

Functional connectivity analysis based on PLV revealed significant differences in network organization between groups (Figure 4d). The high-potential group demonstrated stronger long-range connectivity, whereas the low-potential group showed relatively stronger short-range connectivity patterns. In the theta band, long-range connectivity was significantly enhanced in the high-potential group (*t* = 3.0, $p_{\mathrm{FDR}}=0.04$, Cohen’s *d* = 0.85). Similarly, beta-band long-range connectivity showed a significant increase in the high-potential group (*t* = 3.08, $p_{\mathrm{FDR}}=0.04$, Cohen’s *d* = 0.88). In contrast, the low-potential group exhibited significantly stronger short-range connectivity in the theta band (*t* = 3.5, $p_{\mathrm{FDR}}=0.03$, Cohen’s *d* = 0.63), while a similar but non-significant tendency was observed in the beta band (*t* = 2.68, $p_{\mathrm{FDR}}=0.06$, Cohen’s *d* = 0.34). These findings indicate that high-potential learners rely more on distributed neural communication, whereas low-potential learners may depend on localized processing strategies with reduced large-scale integration.

### 5.3. Functional Brain Connectivity and PLV Synchronization Comparison

**Figure 5:**
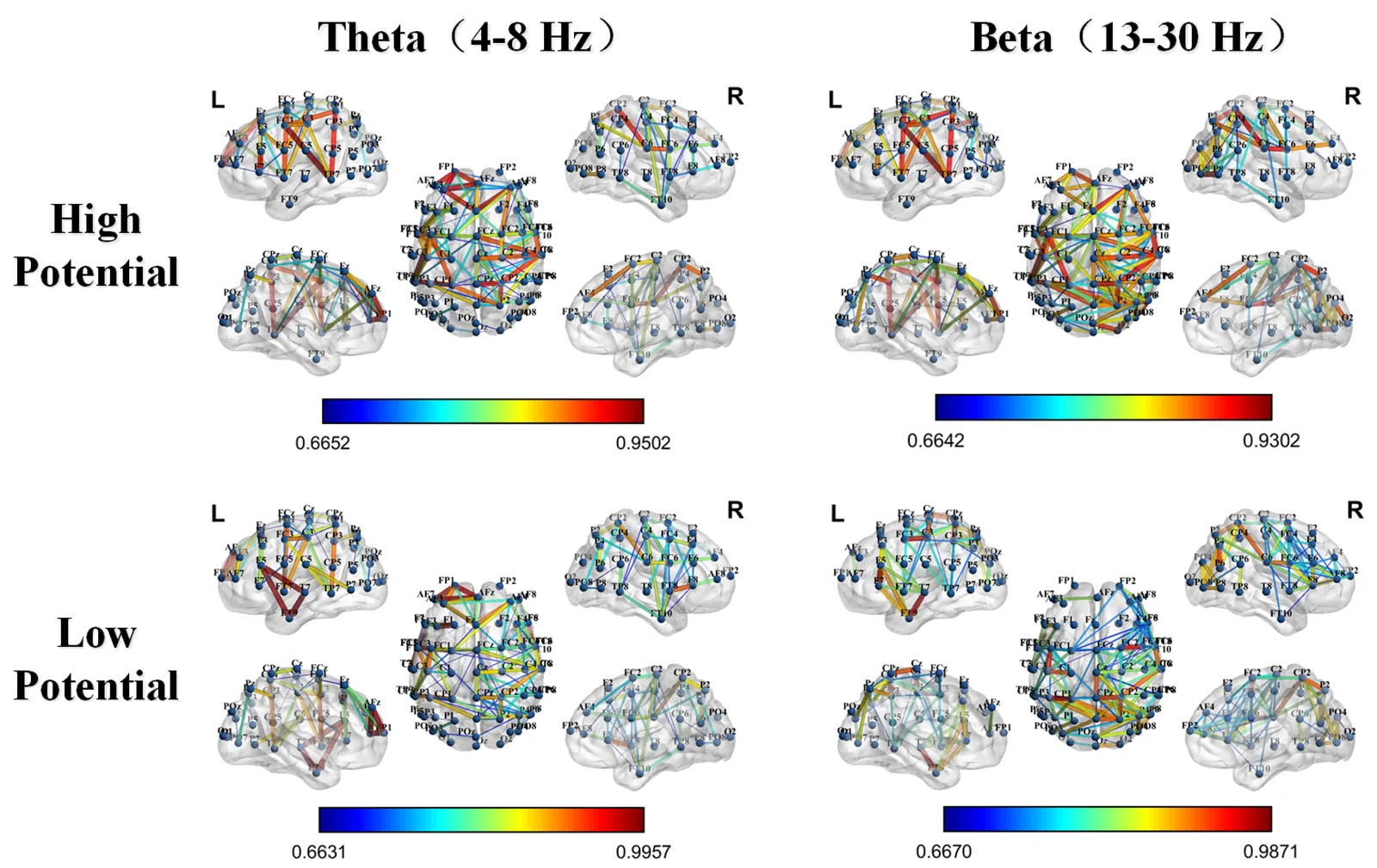
PLV-based functional connectivity patterns between high- and low-potential groups. The color of each edge represents the PLV value, with blue indicating lower phase synchronization and red indicating higher phase synchronization. The color bars indicate the range of PLV values across all displayed connections within each frequency band.

To investigate the neural basis underlying differences in learning potential during English reading comprehension, we compared PLV-based functional connectivity between high- and low-potential groups. Representative theta (4–8 Hz) and beta (13–30 Hz) topological networks are shown in Figure 5.

In the theta band, high-potential participants showed stronger and more extensive connectivity across both hemispheres, particularly among prefrontal, parietal, and central regions. This dense long-range coupling suggests enhanced information integration and cognitive control. In contrast, low-potential participants exhibited sparse and localized theta connectivity, reflecting limited cross-regional communication.

In the beta band, the high-potential group showed higher connectivity among parietal, occipital, temporal, and frontal regions, suggesting stronger linguistic and integrative processing. Conversely, the low-potential group showed weaker and more fragmented beta connectivity, especially in prefrontal-parietal and temporal-occipital circuits. Overall, high-potential individuals displayed a “high-integration, high-synchrony” network profile, whereas low-potential individuals showed diminished functional connectivity and neural synchrony. These findings support the view that learning potential is underpinned by the efficiency and integration of brain networks (Bressler & Menon, 2010).

**Figure 6:**
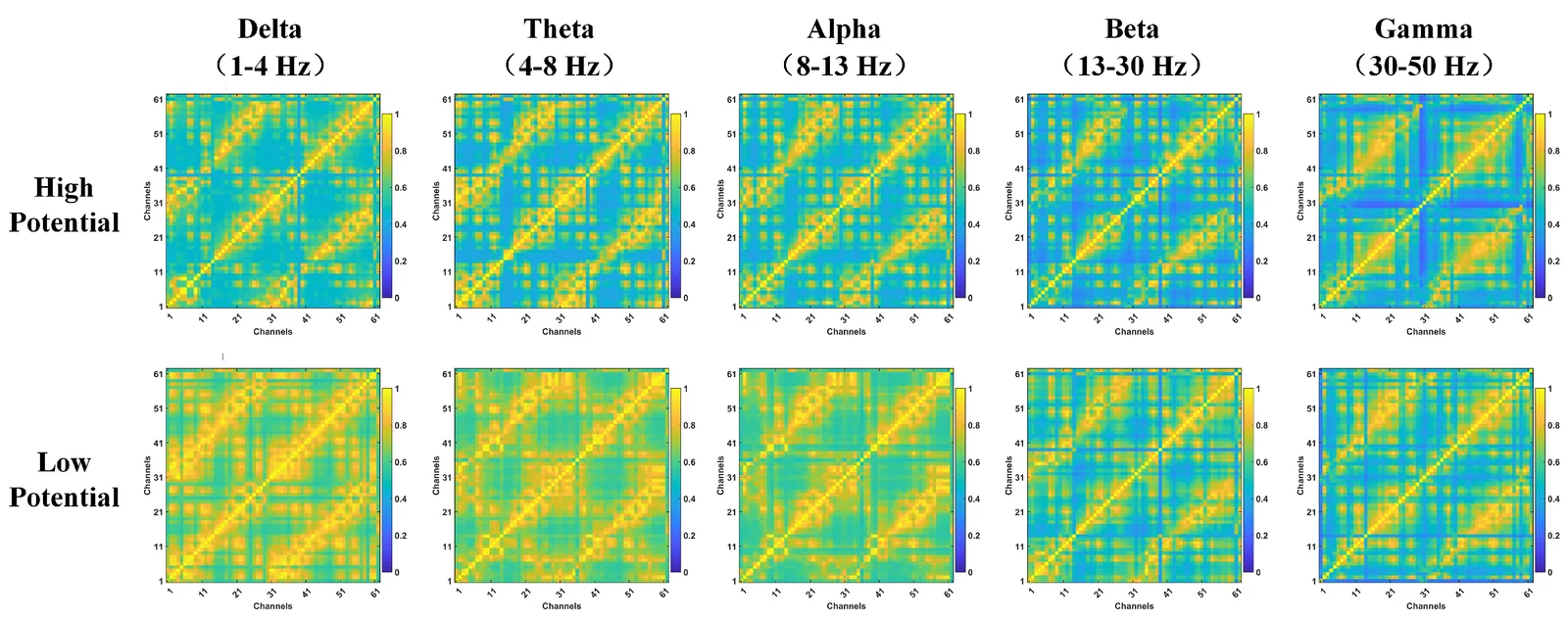
Group-averaged PLV matrices of the high- and low-potential groups across five frequency bands (Delta: 1–4 Hz, Theta: 4–8 Hz, Alpha: 8–13 Hz, Beta: 13–30 Hz, and Gamma: 30–50 Hz). Each matrix element represents the phase-locking value (PLV) between a pair of EEG channels. The color scale ranges from 0 to 1, where lower values (blue) indicate weaker phase synchronization and higher values (yellow) indicate stronger synchronization.

To further examine frequency-specific neural coordination patterns, PLV-based functional connectivity matrices were constructed across five canonical EEG bands (Figure 6). Across all frequencies, the high-potential group showed stronger PLV synchrony and more organized inter-regional connectivity, whereas the low-potential group displayed scattered and irregular synchrony.

Specifically, delta-band connectivity in the high-potential group showed stronger long-range synchronization between prefrontal and central regions, while theta-band connectivity showed enhanced interhemispheric coordination. Alpha-band connectivity was more focused and bilateral, linking occipital-parietal and frontal regions. In the beta band, connectivity extended from prefrontal to temporal and occipital regions, indicating recruitment of large-scale language networks. Gamma-band connectivity was dense and modular, forming hubs in frontal-temporal and temporo-occipital regions. In contrast, the low-potential group generally showed weaker, more localized, and less organized connectivity across these bands, suggesting reduced coordination and network stability.

Together, these results indicate that high learning potential is characterized by coherent, frequency-specific enhancements in large-scale brain connectivity, supporting the integration of cognitive, linguistic, and attentional processes.

### 5.4. SHAP-Based Feature Importance Analysis: Identifying Biomarkers for Learning Potential Classification

To elucidate the contributions of EEG features in classifying students with high and low learning potential, we selected the three most effective feature types—PLV, PSD, and DE—and used them as inputs to a ResNet-18-based classification model. The SHAP framework was employed to quantify the contribution of each feature to the model output decision (Van den Broeck et al., 2022).

**Figure 7:**
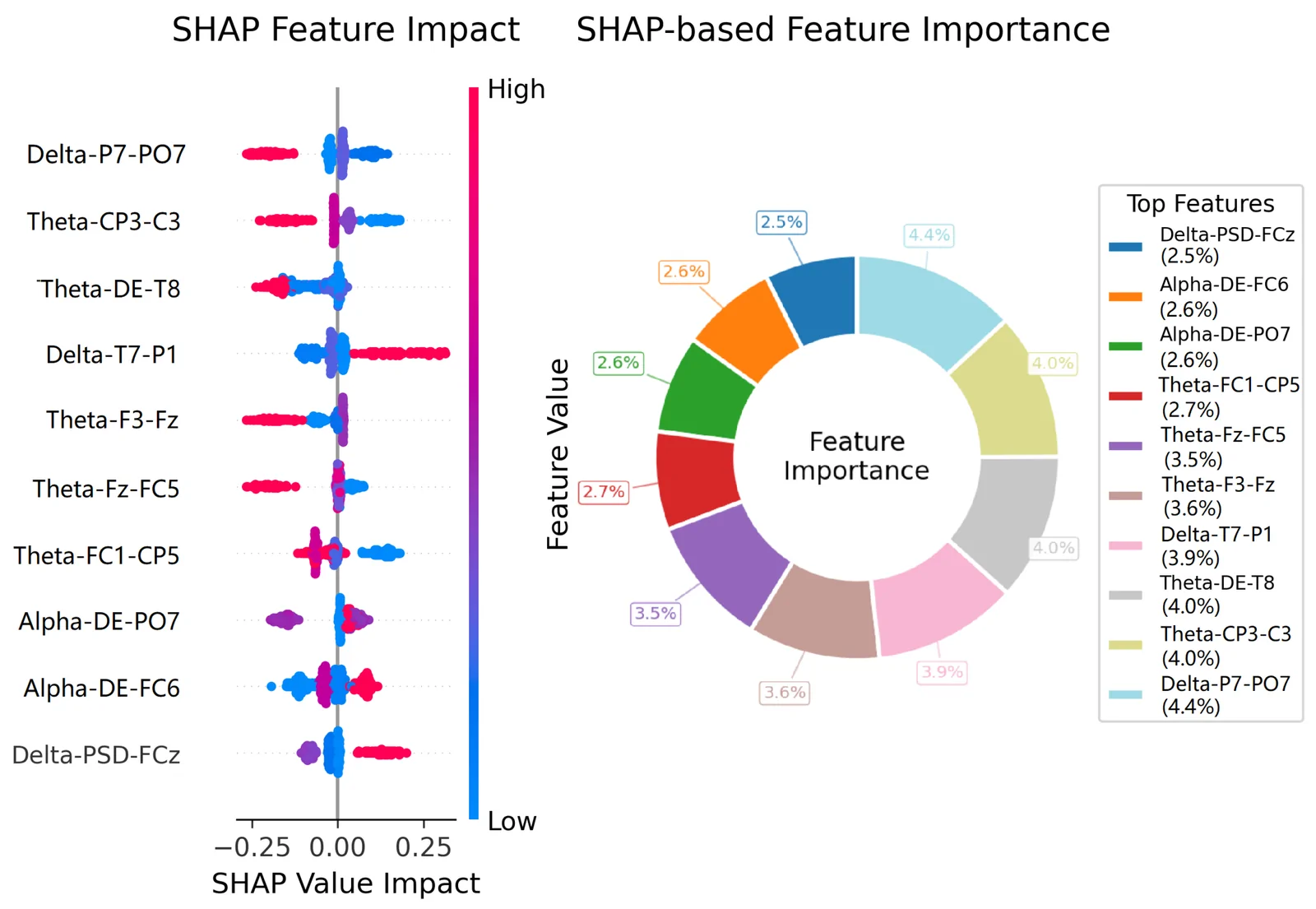
SHAP-based interpretation of the multimodal EEG classification model. SHAP summary plot showing the impact of the ten most important EEG features on model predictions. Each point represents one sample, with the horizontal axis indicating the SHAP value. Positive SHAP values contribute to the high-potential prediction, whereas negative values contribute to the low-potential prediction. Point colors represent the normalized feature values, with blue indicating lower feature values and red indicating higher feature values.

As shown in Figure 7, PLV features in the delta and theta bands emerged as the most influential across all input modalities. In particular, Delta-P7-PO7 and Theta-CP3-C3 exhibited the highest SHAP values (all exceeding 4.0%), indicating that low-frequency phase synchronization provided important information for distinguishing the two learning-potential groups. PLV values from parieto-occipital, central, and frontal midline regions were positively associated with high-potential predictions, suggesting that stronger inter-regional phase synchrony was associated with cognitive processing patterns related to high-potential classification. Although DE and PSD features showed lower overall SHAP contributions than PLV, several features, including Theta-DE-T8, Alpha-DE-FC6, and Delta-PSD-FCz, were still assigned notable importance, reflecting the contribution of regional neural activity and nonlinear complexity features to model decisions during reading tasks.

The SHAP color distribution further showed that high feature values (red) were associated with positive SHAP contributions toward the “high-potential” prediction, whereas low values (blue) were associated with the “low-potential” prediction. High-frequency features, such as Gamma-band features, exhibited limited importance, indicating that low-frequency functional coupling features provided stronger discriminative information for the classification model.

In summary, the SHAP analysis was consistent with the PLV-based connectivity results, showing that high-potential individuals exhibited stronger fronto-parietal and fronto-occipital phase synchronization. These findings suggest that low-frequency synchrony patterns represent important EEG features contributing to learning-potential classification and may serve as potential neural correlates associated with different dynamic learning profiles.

### 5.5. Optimal Brain Regions and Functional Connectivity

To clarify the spatial and connectivity-level contributions of EEG features in distinguishing individuals with high and low LPS groups, we conducted SHAP-based interpretability analysis at two levels: electrode-level features and functional connectivity features.

**Figure 8:**
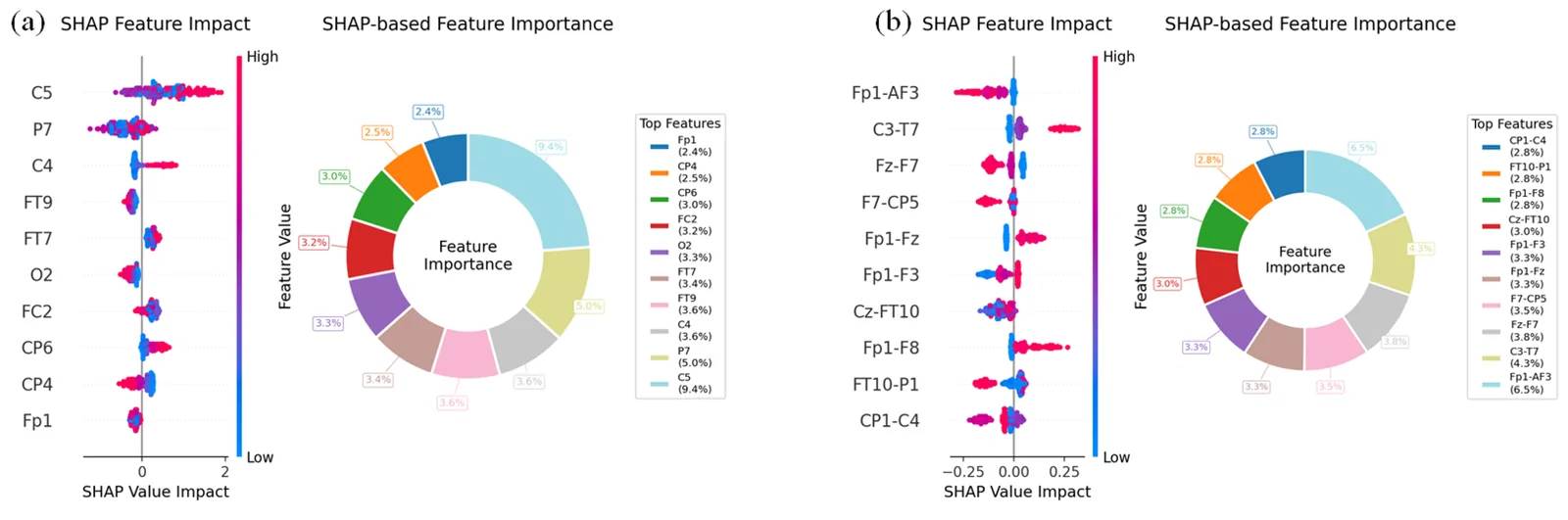
The SHAP importance map for learning potential task. (**a**) Electrode-level SHAP analysis showing the top ten EEG channels contributing to the classification of high and low learning potential; (**b**) Functional connectivity-level SHAP analysis illustrating the most influential inter-regional links derived from the PLV matrix.

As illustrated in Figure 8a, electrodes located in central, parietal, and prefrontal regions exhibited the highest SHAP values, indicating their important contribution to the model classification. Specifically, channels such as C5, P7, C4, and FT9 were assigned high importance by the model. These electrodes are mainly distributed across cortical areas involved in executive control, working memory, and attentional processing. Prefrontal electrodes such as FP1 also showed high SHAP values, suggesting that activity patterns in these regions provided important discriminative information for distinguishing different LPS-defined groups (Valk et al., 2016).

Furthermore, SHAP analysis was conducted on functional connectivity features derived from the PLV matrix, as shown in Figure 8b. The results revealed that bilateral prefrontal, fronto-parietal, and central-occipital connections made substantial contributions to the model predictions. Specifically, short-range intra-prefrontal connections (e.g., FP1-AF3) and long-range left temporal-parietal connections (e.g., C3-T7) were among the most influential features for distinguishing high- and low-potential groups. These findings suggest that connectivity patterns involving frontal, parietal, and temporal regions provided important information for model-based discrimination during reading tasks.

Compared with the high-potential group, the low-potential group showed different connectivity patterns in the identified PLV features, suggesting reduced efficiency of large-scale neural coordination during task engagement. However, these SHAP-derived features should be interpreted as model-associated EEG representations rather than direct evidence of underlying biological mechanisms. Together, these findings indicate that fronto-parietal and temporo-parietal synchronizations represent important EEG connectivity features contributing to LPS-group classification, and may serve as potential neural correlates associated with individual differences in dynamic learning performance (Wang et al., 2019).

## 6. Discussion

This study developed an EEG-based framework to characterize individual differences in task-specific dynamic learning performance during English reading tasks. By integrating EEG-derived features, functional connectivity, deep learning, and SHAP-based interpretability, the framework provides a neurophysiologically informed approach for investigating neural representations associated with LPS-defined learning profiles beyond traditional behavioral and psychometric evaluations.

### 6.1. Neurophysiological Interpretation of Learning Potential

Students with high LPS values exhibited stronger global synchrony in PLV-based functional connectivity networks, particularly among prefrontal, parietal, and central cortical regions. Rather than showing uniform whole-brain enhancement, these connections demonstrated region-specific strengthening within networks supporting attention, working memory, and language-related processing. This pattern aligns with the Efficient Brain Network Hypothesis, which suggests that individuals with higher cognitive abilities may rely on more optimized connectivity architectures (Langer et al., 2012).

In contrast, although the low-potential group showed some local connectivity enhancements, such as short-range links in the left central region, their networks exhibited relatively weaker long-range high-synchrony connections. This pattern suggests reduced large-scale neural coordination during complex cognitive tasks requiring distributed information integration (Guan et al., 2022). However, these connectivity differences should be interpreted as neural patterns associated with different LPS-defined groups rather than direct evidence of distinct learning mechanisms.

### 6.2. Consistency Between Model Interpretability and EEG-Based Neural Patterns

SHAP-based interpretation further supported the interpretability of the proposed framework. At the electrode level, channels such as C5, P7, C4, and FT9 showed high feature importance, indicating that electrophysiological variations in these regions provided discriminative information for distinguishing individual differences during reading. Compared with PLV-based analyses, SHAP interpretation further emphasized left-hemispheric electrodes, such as C5 and P7, suggesting that activity patterns in these regions contributed to model classification and may reflect differences in neural processing strategies between the two LPS-defined groups.

At the functional connectivity level, long-range links such as FP1-AF3, C3-T7, and Fz-F7 showed high SHAP values. These connections mainly involved prefrontal, temporo-parietal, and fronto-central networks, consistent with PLV-based observations and indicating that these connectivity patterns provided important information for distinguishing the two LPS-defined groups (Bashivan et al., 2017). Interhemispheric connections, such as FP1-F8, also showed high importance, suggesting that bilateral connectivity patterns contributed to model-based differentiation of dynamic learning profiles.

Overall, the consistency between PLV-based connectivity patterns and SHAP-based feature importance indicates that the model relied on EEG representations that were also observed in conventional connectivity analysis. These findings suggest that the identified features represent interpretable EEG patterns associated with LPS-group classification rather than directly validated neurophysiological biomarkers. This helps alleviate the black-box nature of deep learning models by providing insights into which EEG features contribute to classification decisions and strengthens the interpretability of EEG-based dynamic learning assessment.

These findings should be interpreted within the context of the current dynamic assessment paradigm. Because the LPS groups were defined according to task-related behavioral performance, the identified EEG representations primarily reflect neural characteristics associated with different LPS-defined learning profiles. Thus, the present results provide neurophysiological evidence for individual differences in dynamic learning performance, rather than an independent validation of learning potential as a stable neural trait. Further validation across independent assessment tasks and longitudinal learning outcomes will be necessary to determine the broader applicability of these neural patterns.

### 6.3. Limitations of the Current Study

Several limitations should be acknowledged. First, the relatively small sample size may limit the generalizability of the deep learning model. Although LOSO-CV, confidence intervals, effect sizes, and FDR correction were employed, formal statistical inference for classification performance, such as permutation testing, was not conducted and should be considered in future studies. Second, the current study focused on an English reading task, and the extent to which the identified EEG patterns generalize to other learning domains remains to be investigated. Third, the current LPS has not yet been directly compared with established dynamic assessment tools, and such comparative validation is needed to further establish its convergent validity. Fourth, the LPS labels were derived from behavioral performance within the same dynamic learning paradigm in which EEG signals were recorded. Although the classifier used only EEG features as input, this design introduces potential circularity between behavioral labeling and EEG-based classification. Therefore, the identified EEG patterns should be interpreted as being associated with LPS-defined learning profiles rather than as an independent neural measure of learning potential. Finally, the present analysis mainly relied on static functional connectivity features. Future work could incorporate dynamic connectivity or directional interaction analyses to further characterize the temporal organization of learning-related neural processes (Shojaie & Fox, 2022).

In summary, this study presents an EEG-based framework integrating multiscale neural features, deep learning, and model explainability to characterize neural representations associated with individual differences in dynamic learning performance. The findings highlight differences in electrode responses and inter-regional coordination between high- and low-potential groups, providing theoretical insights and a methodological foundation for future research on neurocognitive assessment and personalized learning.

## 7. Conclusions

This paper presents an EEG-based Multi-scale Learning Potential Assessment (MS-LPA) framework for comprehensively assessing learning potential during complex reading comprehension tasks. The framework addresses three major limitations of existing approaches: (1) the inability of traditional assessments to capture dynamic process-related information, (2) the lack of neural coordination perspectives for modelling cognitive potential in learning, and (3) the limited interpretability of deep learning models in this domain.

Specifically, MS-LPA achieves three key objectives. First, a continuous dynamic task structure involving reading comprehension, responsive cueing, and error-based revision is designed to induce neurocognitive states across different potential levels. Second, entropy-based and functional connectivity metrics are extracted to characterize both signal complexity and inter-regional neural coordination during learning. Third, SHAP-based interpretability analysis of PLV features within the ResNet-18 model enables the identification of core biomarkers of learning potential, achieving an accuracy of 0.83.

Overall, this study confirms that EEG signals can be leveraged to construct a reliable and explainable assessment system for learning potential. It provides objective neurophysiological evidence for understanding individual differences in cognitive functioning and contributes to interdisciplinary research in neuroeducation. Future work should deploy this framework in real classroom settings and incorporate multimodal physiological signals to further validate its adaptability and practical value.

## Figures and Tables

**Table 1:** Architecture details of the ResNet-18 model.

Layer	Type	Output Size	Kernel/Stride
Input	EEG Input (n×62)	n×62	–
Conv1	Conv2D (64 filters)	n×64	7×7/2
MaxPool	MaxPooling	n/2×64	3×3/2
Conv2_x	2 × BasicBlock (64)	n/4×64	3×3/1
Conv3_x	2 × BasicBlock (128)	n/8×128	3×3/2
Conv4_x	2 × BasicBlock (256)	n/16×256	3×3/2
Conv5_x	2 × BasicBlock (512)	n/32×512	3×3/2
AvgPool	Global Average Pooling	1×512	–
FC	Fully Connected + Softmax	2	–

**Table 2:** Classification performance (ACC and AUC) across frequency bands for different EEG features.

	Delta (1–4 Hz)		Theta (4–8 Hz)		Alpha (8–13 Hz)		Beta (13–30 Hz)		Gamma (30–50 Hz)	
	ACC	AUC	ACC	AUC	ACC	AUC	ACC	AUC	ACC	AUC
Raw	0.61 ± 0.12	0.58 ± 0.1	0.61 ± 0.13	0.59 ± 0.09	0.69 ± 0.15	0.65 ± 0.11	0.58 ± 0.19	0.55 ± 0.15	0.49 ± 0.20	0.48 ± 0.20
DE	0.71 ± 0.08	0.73 ± 0.09	0.77 ± 0.11	0.75 ± 0.12	0.69 ± 0.05	0.68 ± 0.08	0.71 ± 0.12	0.75 ± 0.05	0.71 ± 0.04	0.73 ± 0.08
PSD	0.70 ± 0.10	0.65 ± 0.09	0.71 ± 0.15	0.69 ± 0.13	0.72 ± 0.16	0.78 ± 0.16	0.77 ± 0.11	0.78 ± 0.10	0.73 ± 0.19	0.73 ± 0.17
SE	0.61 ± 0.20	0.55 ± 0.19	0.65 ± 0.18	0.59 ± 0.17	0.68 ± 0.16	0.70 ± 0.15	0.70 ± 0.19	0.71 ± 0.20	0.69 ± 0.13	0.69 ± 0.14
**PLV**	**0.77 ± 0.05**	**0.78 ± 0.08**	**0.82 ± 0.09**	**0.85 ± 0.10**	**0.79 ± 0.11**	**0.82 ± 0.12**	**0.83 ± 0.05**	**0.84 ± 0.05**	**0.80 ± 0.09**	**0.81 ± 0.05**

**Table 3:** Demographic and behavioral characteristics of the high- and low-potential groups.

Variable	High-Potential	Low-Potential
*N*	24	26
Age	22.14±1.85	21.86±2.01
Accuracy	0.73±0.18	0.50±0.25
Reaction Time	0.81±0.05	0.79±0.37
Error Correction	0.50±0.32	0.33±0.28
Final Scores	0.86±0.08	0.66±0.19

## Data Availability

All data generated or analyzed during this study are available from the corresponding author on reasonable request (https://github.com/yzy-zyyang/MS-LPA (accessed on 17 August 2026)).
